# Trash to Treasure: An Up-to-Date Understanding of the Valorization of Seafood By-Products, Targeting the Major Bioactive Compounds

**DOI:** 10.3390/md21090485

**Published:** 2023-09-09

**Authors:** Vikash Chandra Roy, Md. Rakibul Islam, Sultana Sadia, Momota Yeasmin, Jin-Seok Park, Hee-Jeong Lee, Byung-Soo Chun

**Affiliations:** 1 Institute of Food Science, Pukyong National University, 45 Yongso-ro Namgu, Busan 48513, Republic of Korea; 2 Department of Fisheries Technology, Hajee Mohammad Danesh Science and Technology University, Dinajpur 5200, Bangladesh; 3 Department of Food Science and Technology, Pukyong National University, 45 Yongso-ro Namgu, Busan 48513, Republic of Korea; jin1931@pukyong.ac.kr; 4 Department of Food Science and Nutrition, Kyungsung University, Busan 48434, Republic of Korea; leehjeong@ks.ac.kr

**Keywords:** seafood by-product, valorization, bioactive compounds, green extraction methodologies, circular economy

## Abstract

Fishery production is exponentially growing, and its by-products negatively impact industries’ economic and environmental status. The large amount of bioactive micro- and macromolecules in fishery by-products, including lipids, proteins, peptides, amino acids, vitamins, carotenoids, enzymes, collagen, gelatin, chitin, chitosan, and fucoidan, need to be utilized through effective strategies and proper management. Due to the bioactive and healthy compounds in fishery discards, these components can be used as functional food ingredients. Fishery discards have inorganic or organic value to add to or implement in various sectors (such as the agriculture, medical, and pharmaceutical industries). However, the best use of these postharvest raw materials for human welfare remains unelucidated in the scientific community. This review article describes the most useful techniques and methods, such as obtaining proteins and peptides, fatty acids, enzymes, minerals, and carotenoids, as well as collagen, gelatin, and polysaccharides such as chitin–chitosan and fucoidan, to ensure the best use of fishery discards. Marine-derived bioactive compounds have biological activities, such as antioxidant, anticancer, antidiabetic, anti-inflammatory, and antimicrobial activities. These high-value compounds are used in various industrial sectors, such as the food and cosmetic industries, owing to their unique functional and characteristic structures. This study aimed to determine the gap between misused fishery discards and their effects on the environment and create awareness for the complete valorization of fishery discards, targeting a sustainable world.

## 1. Introduction

Presently, a rapidly growing population demands quality food and ingredients as part of their regular consumption preferences. Fish is considered the most accessible source of protein, and protein-based food ingredient materials are cheaper than other sources. In the fishery sector, the amount of fish and fish-related products (crustaceans, fish, mollusks, and others) has increased, reaching 177.8 million tons (MT) in 2020; this includes 157.4 MT directly used for human food and 20.2 MT utilized in nonfood applications [1]. Globally, the annual discarded fish waste was estimated at 9.1 MT, comprising 10.8% of the annual average catches in 2010–2014 [2]. In 2018, fish production was estimated to reach 179 MT, producing nearly 20–23 MT of fishery by-products. Following that, the rapidly expanding aquaculture industry contributed approximately 52% of the fish people consumed [3].

**Figure 1:**
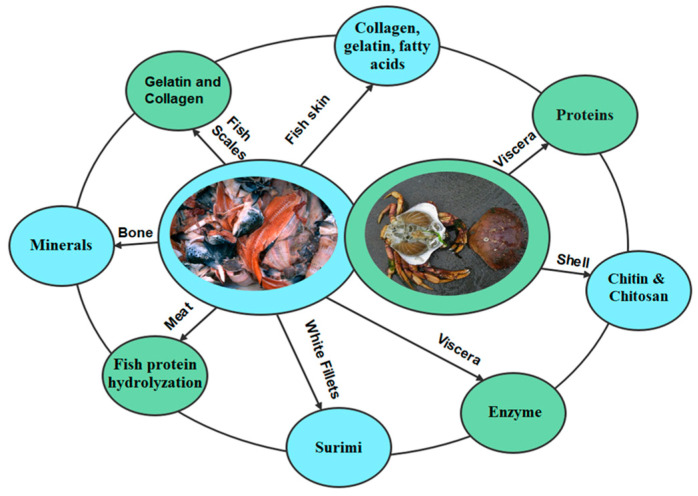
Extraction of various bioactive compounds from different fishery by-products.

Fishing activities in the ocean generate many by-products with economic and environmental impacts [3]. However, the knowledge of the effective valorization of these by-products can help in sustainable management, thus reducing the negative impact. In addition to fishery by-product generation during harvests in marine and coastal waters, a considerable amount is generated from the fish-processing industries and domestic markets. These by-products typically comprise the skin, scales, bone, fins, visceral parts, heads, and shells of various crustaceans [4], which are considered excellent sources of different bioactive compounds [4,5,6,7] (Figure 1).

Various studies have shown the use of fishery by-products as feeds in the poultry and cattle industries [8,9]. Such low-cost applications hinder maximum utilization; hence, combined information on these discards’ availability and potentiality can help create a strategy. Moreover, by 2050, the world population is estimated to be approximately 9.2 billion, requiring effective strategies for a sustainable food supply and a reduction in pollution to protect the environment [10]. The effective utilization of large amounts of fishery by-products can significantly help the food, pharmaceutical, and other industries. Previous studies have proven these by-products to be treasures upon their utilization.

**Figure 2:**
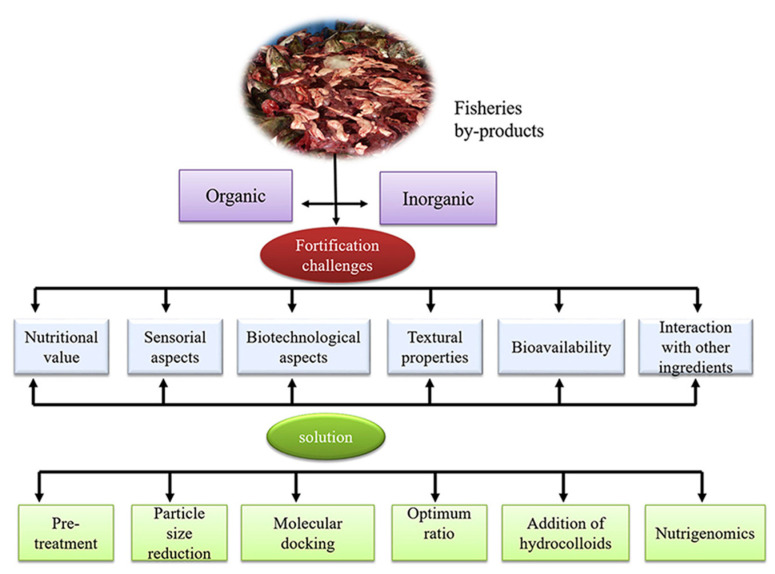
The nutrients from fishery by-products can be used for food fortification. “Adapted with permission from Nawaz et al. [6]. 2020, Elsevier Ltd.”.

Regarding their nutritional values and functional properties, fishery by-products generally contain 15–30% protein, 0–25% crude lipids, 50–80% moisture, and different vitamins and minerals [3,6,11]. Crustacean by-products are a rich source of different macromolecules, including chitin, chitosan [12,13], polyunsaturated fatty acids, and various valuable carotenoids such as astaxanthin [14,15]. The health benefits of these compounds prompt scientists to think of better applications for these by-products (Figure 2).

Thus, this review aimed to summarize recent studies targeting the valorization of fishery by-products. Converting low-value seafood by-products to high-value bioactive compounds is an excellent economic and environmental approach. Reducing the loss of valuable compounds in the by-products can also bring additional benefits and help humanity achieve a sustainable world. Different food, pharmaceutical, and cosmetic industries use bioactive compounds such as gelatin, collagen, carotenoids, and unsaturated fatty acids from fishery sources owing to their good acceptability worldwide. We believe that this review will provide up-to-date information on the valorization of seafood or marine by-products, targeted bioactive compounds, their extraction technologies, and their functional properties.

## 2. Fishery By-Products

According to the fishery dictionary of the United Nations Food and Agricultural Organization (FAO), “fishery by-products can be defined as the proportion of the total organic material of animal origin in the catch, which is thrown away or dumped at sea, for whatever reason. It does not include plant material and postharvest waste such as offal.” Also, it describes bycatch as “the part of a catch of a fishing unit taken incidentally in addition to the target species toward which fishing effort is directed. Some or all of it may be returned to the sea as discards, usually dead or dying” [16]. Table 1 shows the amount of by-products generated by major seafood items.

## 3. Fish By-Products as a Source of Various Bioactive Compounds

### 3.1. Concentrated Seafood Protein

Seafood-generated by-products such as fish heads, skin, bones, scales, fins, and blood contain a high amount of proteins and other bioactive compounds [3,6,11,24]. Generally, proteins in their original forms are tightly attached via different chemical forces, including hydrophobic effects, electrostatic interactions, hydrogen bonds, van der Waals forces, and disulfide bonds [25].

Methods for recovering protein from fishery by-products differ. The most common include fish protein hydrolysates, fish protein isolates, and the extraction of myofibrillar types of protein (surimi).

#### 3.1.1. Fish Protein Hydrolysates

Several methods exist for obtaining protein hydrolysates from fishery by-products. The most common are chemical hydrolysis via acidic or alkaline mediums, bacterial fermentation, and enzymatic hydrolysis [26]. Although chemical hydrolysis has a low cost and rapidly extracts protein compared with enzymatic hydrolysis, many scientists prefer enzymatic hydrolysis due to its several advantages. Using enzymatic hydrolysis, approximately 15–30% of protein is obtainable from fishery by-products [11]. Several studies have reported little control over the consistency of the hydrolyzed products and the variations in the free amino acid profile for the nonspecific breakdown of peptide bonds [26,27]. To hydrolyze fish protein or to turn the protein from fish by-products into peptides or amino acids, combined cleaved proteins are called fish protein hydrolysates. A factor contributing to its interest is the use of industrially appropriate hydrolysis. Because they can recover large amounts of protein, research on them has recently increased [26,28]. Enzymatic protein hydrolysis, which uses endogenous enzymes to cleave peptide bonds in amino acids, has recently been considered an alternative to traditional chemical hydrolysis. The protein obtained from fishery discards using enzyme hydrolysis varies significantly depending on the sex, age, diet, and even season of different species [26]. Table 2 shows the proteins extracted from fishery by-products using different methods and extraction processes.

#### 3.1.2. Fish Protein Isolates

Seafood protein can be isolated using the isoelectric solubilization method, commonly known as the pH-shifting method. This isoelectric precipitation method involves three steps. First, the protein from the marine by-products is solubilized at a higher pH value (primary condition). Then, the solubilized protein is turned to a neutral pH to disrupt the cellular membrane and separate unwanted molecules via centrifugation, and, finally, the isolated protein can precipitate at the isoelectric point [28]. Tang et al. [25] reported that protein extraction using the pH-shifting method showed better gelling, water-holding, emulsification, and oil-holding properties. The nutritional value of the extracted protein highly depends on the presence of essential amino acids [29]. Several studies have shown that protein obtained using the pH-shifting method resulted in a high amount of essential amino acids compared with other extraction methods [25,29,30].

#### 3.1.3. Surimi

Myofibrillar types of protein obtained using the mechanical deboning and washing of the fish process (fish flesh) are referred to as surimi. While preparing surimi, except for the myofibrillar protein, all other compounds, such as sarcoplasmic protein, connective tissues, and crude lipids, are removed via repeated washing and dewatering processes. It is generally prepared from low-cost lean fish, bycatch species, and other marine by-products. Surimi is an excellent concentrated protein source and a popular intermediate raw material for preparing popular seafood products such as kamaboko, fish or crab analog, and sausage [3,31,32].

**Table 2:** Protein hydrolysates obtained from different fishery products, their extracting agents, and the functionalities of the obtained proteins.

Fish Species	By-Products	Extraction Agents (Enzymes)	Properties/Activities	References
Channel catfish (*Ictalurus punctatus*)	Frames and heads	Ficin, neutrase, protamex, papain, novo-proD, thermolysin, alcalase, and bromelain	Protease reaction kinetics showed that ficin was the most efficient to hydrolyze catfish proteins, and the foaming and emulsifying properties of the protein hydrolysates were observed.	[33]
Striped catfish (*Pangasianodon Hypophthalmus*)	Viscera	Enzymatic (pepsin and papain) and chemical process (e.g., NaOH, HCl)	The spray-dried and enzymatically extracted hydrolysate had lower turbidity with increasing pH; the lowest solubility, foaming capacity, and stability were observed at pH 5.0.	[34]
Small-spotted catshark (*Scyliorhinus canicula*)	By-products of muscle	Enzymatic hydrolysis (protamex, esperase, and alcalase)	Protein hydrolysates obtained via enzymatic hydrolysis showed strong antihypertensive and antioxidant properties.	[35]
Bluefin leatherjacket (*Navodon* *Septentrionalis*)	Heads	Enzymatic hydrolysis (papain)	Protein hydrolysates showed antioxidant properties.	[36]
Sardinella (*Sardinella aurita*)	Viscera and heads	Enzymatic hydrolysis (microbial proteases)	Protein hydrolysates showed antioxidant properties.	[37]
Rainbow trout (*Oncorhynchus mykiss*)	Fins, heads, backbone, and viscera	Enzymatic hydrolysis (alcalase)	Protein hydrolysates showed antioxidant properties.	[38]
Red tilapia (*Oreochromis* spp.)	Viscera	Enzymatic hydrolysis (alcalase)	Peptide fractionation was performed using ultrafiltration, and the <1 kDa fraction (FRTVH-V) expressed the highest iron-binding capacity.	[39]
Anchovies (*Engraulis encrasicolus*)	Viscera	Enzymatic hydrolysis (alcalase, flavourzyme, and protamex)	Protein hydrolysates showed activities in in vitro and in vivo model biological activities by decreasing the severity of oxidative stress.	[40]
Bluefin leatherjacket (*Navodon* *Septentrionalis*)	Skins	Enzymatic hydrolysis (alcalase, trypsin, papain, neutrase, pepsin, and flavourzyme)	The antioxidant activities of peptides were evaluated with three radical scavenging and lipid peroxidation inhibition assays.	[41]
Australian rock lobster (*Jasus edwardsii*)	Shells	Enzymatic hydrolysis (alcalase)	The protein hydrolysate produced by this study had excellent functionality (solubility 91.7%, water absorption 32%, oil absorption 2.3 mL/g, foaming 51.3%, emulsification 91.3%) and high nutritional quality (34% essential amino acids, 45.4 mg/g arginine, lysine/arginine ratio 0.69) with potential applications for the food industry.	[42]
Horse mackerel (*Magalaspis cordyla*) and croaker (*Otolithes ruber*)	Skins	Enzymatic hydrolysis (trypsin, α-chymotrypsin, and pepsin)	Peptides presented in protein hydrolysates exhibited higher activity against polyunsaturated fatty acid peroxidation than the natural antioxidant α-tocopherol.	[43]
Atlantic salmon (*Salmo salar*)	Backbones and heads	Enzymatic hydrolysis (protex 7 L, promod 671 L, and alcalase 2.4 L)	Chemical, surface activity, and sensory properties were shown.	[44]
Serra Spanish mackerel (*Scomberomorus Brasiliensis*)	Scales crushed and bones	Enzymatic hydrolysis (flavourzyme, and alcalase)	Protein hydrolysate showed better technological performance by stabilizing emulsions and retaining oil, and they could be added to emulsified products, improving their technological and sensory aspects.	[45]
Black scabbardfish (*Aphanopus carbo*)	Frames, heads, skin, trimming, and viscera	Enzymatic hydrolysis (protamex)	The protein hydrolysates presented some antioxidant activity, which increased with increasing degree of hydrolysis.	[46]
Anchovy	Fish sauce by-product (FSB)	Enzymatic hydrolysis (proteinase K)	The low-molecular-weight FSB fraction contained potent antioxidative molecules, which were identified as PQLLLLLL and LLLLLLL.	[47]
Atlantic holothurian (*Cucumaria frondosa*)	Internal organs and aqua-pharyngeal bulb	Enzymatic hydrolysis (proteases)	Enzymatic hydrolysates extracted from by-products of the marine invertebrates were demonstrated as active against HSV-1 (Herpes Simplex virus 1).	[48]
Skipjack tuna (*Katsuwonus pelamis*)	Head and bone	In-vitro gastrointestinal (GI) digestion method	Protein hydrolysates can be applied in health care products as antioxidant agents.	[49,50]
Chinese sturgeon (*Acipenser sinensis*)	Whole body	Enzymatic hydrolysis (papain and alcalase 2.4 L)	Hydrolysates can be used as natural antioxidant substitutes in pharmaceuticals and food products.	[51]
Monkfish (*Lophius piscatorius*)	By-products (head and viscera)	Enzymatic hydrolysis (alcalase)	Protein hydrolysates showed antioxidant and antihypertensive activities.	[52]
Salmon	Viscera	Enzymatic hydrolysis (papain, alcalase, and autolysis process)	The results showed that the obtained protein-rich hydrolysates from fish industries are a promising alternative for expensive nitrogen sources that are commonly used for fermenting yeasts.	[53]
Australian rock lobster	Heads	Chemical process and enzymatic hydrolysis (alcalase)	The results of this study demonstrated the potential value of lobster protein hydrolysates used as a safe emulsifier with significant nutritional value for the food industry.	[54]
Eel (*Conger myriaster*)	Skin	Subcritical water hydrolysis	Strong antioxidant activities.	[55]
Yellow corvina (*Larimichthys polyactis*)	Head and viscera	Subcritical water hydrolysis	Protein hydrolysates showed excellent antioxidant, antidiabetic, and anticancer activity.	[4]
Comb Penshell (*Atrina pectinata*)	Viscera	Subcritical water hydrolysis	Protein hydrolysates showed good antioxidant and antihypertensive activity.	[56]

### 3.2. Extraction and Biofunctionality of Peptides from Marine By-Products

Bioactive peptides, generally inactive protein fragments obtained by the action of enzymes, regulate the body’s receptor and physiological functions, including antihypertensive, antibacterial, antifungal, antioxidant, antiproliferative, immunomodulating, anticoagulant, and antiviral activities [3]. Peptides obtained from seafood by-products also showed angiotensin enzyme (ACE)-inhibition activity, calcium and opioid binding inhibition, and hemolytic properties [57]. Marine fishery discards have been found to be good sources of bioactive peptides [58]. Table 3 shows recent studies on peptide extraction. Fishery discards (usually skin, fin, and head) are rich sources of collagen and gelatin. Fish gelatins contain hydrophobic amino acids (hydroxyproline, valine, glycine, proline, and alanine) with a certain range of peptides with strong antioxidant activities [3,59]. Lassoued et al. [60] showed that the antioxidant activities of ray skin (thornback) were mainly due to the presence of hydrophobic amino acids.

There are different technologies for extracting bioactive peptides from marine biomass. The traditional methods are enzymatic [37,43,48,51] and fermentation hydrolysis [47]. Enzymatic hydrolysis is mostly preferred and effective due to its high specificity, and this methodology does not require any toxic chemicals. The main commercial enzymes for producing bioactive peptides are alcalase, flavorzyme, neutrase, pepsin, trypsin, and papain [36,37,43,49,50,51]. Subcritical water hydrolysis has attracted attention as a green extraction methodology for extracting peptides from marine biomass [55,56].

Purifying peptides from hydrolysates is costly and considered economically unprofitable. Therefore, there is limited research on the purification of peptides from protein hydrolysates. Several researchers have worked on crude protein hydrolysates from seafood discards without purifying peptides [10]. Although seafood peptides have high antioxidant properties, their application is limited in the food industry due to their fishy odors and taste, which is unacceptable to some consumers [61]. Therefore, a large scope exists to develop the taste of protein hydrolysates containing bioactive peptides.

There are various other peptides, along with antioxidant peptides, including antimicrobial and ACE-inhibitory peptides. Antimicrobial peptides are considered the building blocks of animal defense systems [3]. Fish-originated antimicrobial peptides are positively charged and contain a higher amount of hydrophobic amino acids than those obtained from terrestrial animals [62]. Antimicrobial peptides from snow crab and Atlantic crab by-products showed potent inhibitory activity against Gram-positive and -negative bacteria [63]. Researchers intend to develop modern antibiotics and food preservation agents from marine-derived antimicrobial peptides.

Due to their biofunctional abilities, peptides derived from marine sources are gaining popularity in different industries. It is assumed that synthetic drugs will be replaced by marine peptides with similar efficacy but lower or no adverse effects [64].

**Table 3:** Peptides from various fishery by-product derivatives, some examples of which are the studies below.

Fish Species	Body Parts	Extraction (Hydrolysis) Agents (Enzymes)	Properties/Activities	References
Tilapia	Skin gelatin	Pepsin and pancreatin	ACE-inhibitory activity.	[65]
Rainbow trout (*Oncorhynchus mykiss*)	Skin	Flavourzyme, alcalase, and ultrafiltration method	Anticancer, antioxidant properties present in fractions and non-fraction peptides.	[66]
Pacific cod (*Gadus macrocephalus*)	Skin gelatin	Alcalase, papain, trypsin, neutrase, and pepsin	ACE-inhibitory activity.	[67]
Cod (*Gadus morhua*)	Frames	Trypsin, pepsin, and those chymotrypsin combinations	Antioxidant properties.	[68]
Atlantic rock crab (*Cancer irroratus*)	By-products	Proteolytic enzyme action on processing	Antibacterial activity.	[63]
Smooth hound (*Mustelus mustelus*)	Viscera wastes	Proteases (commercial), endogenous enzymes, and those combinations	ACE-inhibitory, antimicrobial, and antioxidant activity.	[62]
Codfish blood and sardine	Cooking-water wastes	Membrane ultrafiltration	The peptide fractions from codfish blood exhibited the highest ABTS+ and ORAC values. Peptide fractions from sardine wastewater were capable of inhibiting *Escherichia coli* growth.	[69]
Threadfin breams (*Nemipterus japonicus*)	Frames	Plant proteases (bromelain and papain)	Antioxidant properties (2,2 diphenyl-1-picrylhydrazyl [DPPH] radical scavenging activity, ferric-reducing power, and lipid peroxidation inhibition) of hydrolysates increased with an increase in the degree of hydrolysis.	[70]
Shortfin scad (*Decapterus Macrosoma*)	Bones	Alcalase	Obtained peptides showed angiotensin I-converting enzyme (ACE)-inhibitory activity.	[71]
Northern shrimp (*Pandalus borealis*)	By-products	Papain, protamex, trypsin, flavourzyme, and alcalase	Antioxidant and ACE-inhibitory activity. The results of this research suggested that the high-molecular-weight enzymatic hydrolysate derived from shrimp can be used to control oxidative stress and prevent hypertension.	[72]
Catfish (*Ictalurus punctatus*)	Bone frames and heads	Proteases	The emulsifying and foaming properties and stability of selected hydrolysates were evaluated.	[33]
Skate (*Raja porosa*)	Cartilage	Chromatography and ultrafiltration	The result suggested that the isolated peptides have excellent antioxidant properties.	[73]
Grass carp (*Ctenopharyngodon idella*)	Skin	Alcalase	Novel peptides isolated from grass carp skin possess potent antioxidant activities and might be used for food preservation and medicinal purposes.	[74]
Lizardfish (*Synodus macrops*)	Scale gelatin	Trypsin, papain, bromelain, chymotrypsin, and alcalase	ACE-inhibitory peptides derived from scale gelatin have the potential to be used as healthy ACE-inhibiting drug raw materials.	[75]
Pacific cod (*G. macrocephalus*)	Skin gelatin	Pepsin	Extracted peptides showed potent ACE inhibition with IC50 values of 6.9 and 14.5 μM.	[67]
Anchovy (*Engraulis japonicas*)	Cooking-water wastes	Protamex	Purified antimicrobial activity with no hemolytic activity up to a concentration of 512 μg/mL.	[76]
Thornback ray (*Raja clavata*)	Skin gelatin	Alcalase	ACE-inhibitory activity.	[60]
Seabass (*Lates calcarifer*)	Skin gelatin	Alcalase	Peptides prepared from seabass skin showed good antioxidant activity.	[77]
Atlantic salmon (*Salmo salar)*	Trimming	Alcalase 2.4 L, flavourzyme 500 L, Corolase PP, and Promod 144 MG	Bioactive peptides displayed good DPP-IV and ACE inhibitory and antioxidant activity.	[78]
Skipjack tuna (*Katsuwonus pelamis*)	Roe	Flavourzyme	Four peptides among the fifteen extracted peptides showed remarkable ACE-inhibitory activity.	[79]
Chinese sturgeon (*Acipenser sinensis)*	Whole body	Papain and alcalase 2.4 L	The fractions and purified peptides can be used as natural antioxidant substitutes in pharmaceuticals and food products.	[51]
Atlantic sea cucumber (*Cucumaria frondosa*)	Whole body	Alcalase and trypsin	Generated peptides inhibited MPO (a mediator and marker of in vivo oxidative stress) with predicted molecular interactions.	[80]
Antarctic krill (*Euphausia superba*)	By-products	Trypsin	The preparation process of Antarctic krill peptides-zinc chelate was optimized. Chelate showed excellent stability against various pH and gastrointestinal digestion.	[81]
Squid (*Dosidicus gigas*)	By-products	Protease XIV and ultrafiltration (UFI)	Peptide fractions obtained after UFI had higher antioxidant and antimutagenic activities, but the antiproliferative activity did not improve after UFI.	[82]
Bigeye tuna	Skin	Subcritical water	Peptides obtained via subcritical water hydrolysis showed high antioxidant and antimicrobial activity.	[83]
Skipjack tuna (*Katsuwonus pelamis*)	Skin	Trypsin, neutrase, papain, pepsin, and alcalase	The antioxidant peptides extracted in this study can act as active ingredients in preventing UVA injury.	[84]
Skipjack tuna (*Katsuwonus pelamis*)	Milts	Trypsin, neutrase, papain, pepsin, and alcalase	Bioactive peptides displayed significant protection to HUVECs against H_2_O_2_ damage by increasing antioxidase levels.	[85]
Sturgeon (*Acipenser ruthenus*)	Spermary	Papain	Extracted peptides change the permeability of the microbial cell membranes and may exert antimicrobial activity by inhibiting the metabolic process of the nucleic acids.	[86]
Siberian sturgeon (*Acipenserbaerii*)	Cartilage	Alcalase, papain, trypsin, flavourzyme, and pepsin	The extracted peptides displayed significant cytoprotection on HUVECs against H_2_O_2_ injury.	[87]
Sea intestine (*Urechis unicinctus*)	Viscera	Papain, trypsin, and alkaline protease	Extracted peptides exhibited strong antioxidant activity.	[88]

### 3.3. Fishery Discards as a Source of Lipids

Marine fishery discards are considered an excellent source of edible lipids due to the presence of health-beneficial polyunsaturated fatty acids (PUFAs). Most edible oils are soluble in nonpolar solvents because of their nonpolar behavior [89]. Lipids include triglycerides, waxes, alcohols, cholesterol, phospholipids, and free fatty acids [90].

Fish oil is a significant source of PUFAs in the human diet [3]. Eicosapentaenoic acid (EPA) and docosahexaenoic fatty acid (DHA) are among the health-beneficial PUFAs available in fishery by-products, which are highly demanded in the market [23]. Several studies have reported that PUFAs are helpful for the brain development of infants during pregnancy, reducing the risk of cardiovascular disease in humans, maintaining blood pressure, and improving myocardial activity [3,6,11,90,91].

**Figure 3:**
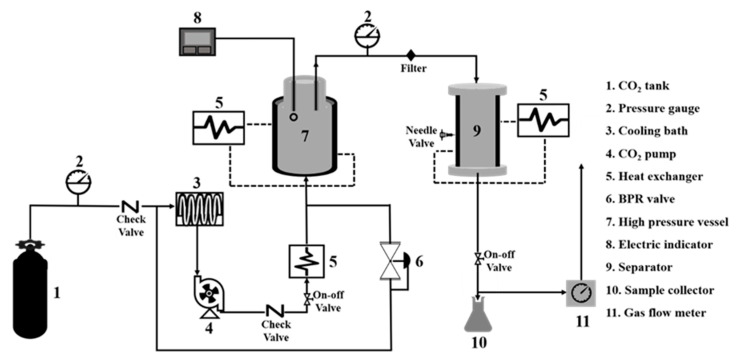
Diagram of supercritical carbon dioxide extraction for extracting high-quality oils from marine resources. This is a promising green technology for extracting fish oil. This figure has been adapted from our previously published article by Roy et al. [23].

Several methods have been applied to extract lipids from fishery resources. Among them, traditional methods such as solvent extraction require high energy and organic solvents, negatively affecting the environment [23]. Hence, several green extraction methods have recently been applied to obtain high-quality oil from fishery discards. The most common green extraction methods are supercritical carbon dioxide (SC-CO_2_) extraction [92], microwave-assisted hydrolysis, and enzymatic hydrolysis [89,92]. SC-CO_2_ is a promising technology for extracting high-quality lipids from fishery discards. This extraction method can easily regulate the extraction of specific fatty acids by controlling the extraction pressure and temperature. A significant advantage of this extraction method is that it does not require any further purification of the oil after extraction. Figure 3 shows a diagram of a typical SC-CO_2_ plant for extracting oils from fishery discards. Table 4 shows the extraction methods of edible oils from seafood discards and their yields, as well as PUFA content.

Seafood is an important source of various unsaturated fatty acids. Omega-3 is a unique unsaturated fatty acid that plays an essential role in the regulation of various biological functions in the human body [93]. DHA is a major structural component of human retinal photoreceptors and helps maintain a healthy visual system [94]. Several studies have shown that PUFAs are essential for developing brain and nerve cells along with the eyes of fetuses and infants [95,96]. Omega-3 fatty acids can also regulate gene expression, cell membrane composition, and eicosanoid production [97]. EPA acts as a precursor of eicosanoids and plays a vital role in hormonal activities. Unsaturated fatty acids can serve as suppressors of triacylglycerol and fatty acid synthesis, thus reducing the risk factors for cardiovascular diseases [94].

The health-beneficial activities of marine-driven PUFAs have high market value. Due to the high demand, PUFA-fortified products are widely available worldwide. Products fortified with fish, krill, and algal oils are popular due to their functionalities [98,99]. Given the oxidative vulnerability of PUFAs, different strategies are necessary to extend their shelf life. However, several antioxidative agents are being used to extend fish oil’s shelf life in the final products. However, several modern technologies, like PUFA microencapsulation by various carrier materials, can be applied, and this new stabilization technique is continuously progressing [100].

**Table 4:** Extraction of high-quality lipids from by-products of different seafood species.

Fish Species	Body Parts	Extraction Methods	Yield of Oil/PUFA Content (%)	References
Seabass (*Dicentrarhus labrax*), bluefin tuna (*Thunnus thynnus)*, and gilthead seabream (*Sparus aurata*)	Liver (bluefin tuna), gills and heads (seabass), guts (seabream)	Raw materials were ground and cooked at 95 °C temperature for 12 min. Then, the materials were pressed with an expeller screw and separated oil, water, and dry matter via centrifugation (at 4200 rpm).	Lipid content: 27 ± 3; PUFA content of tuna by-product: 38 ± 7; tuna; liver: 35 ± 6; cod liver: 34 ± 0.3; sardine oil: 36 ± 3; seabass oil: 30 ± 0.2.	[101]
Rohu (*Labeo rohita*)	Heads	Enzymatic treatment with protamex (1:100 *w*/*w*), with microwave (MW), ultrasound (US), and heat pretreatment (HT).	Crude lipid obtained with MW: 60.45–69.75; US: 58.74–68.08; HT: 31.98–39.03. PUFA content with MW: 37.51 ± 0.53, US: 39.28 ± 0.33, HT: 38.31 ± 0.17.	[102]
Yellowtail fish (*Seriola quinqueradiata*)	Viscera	SC-CO_2_ extraction method and solvent extraction methods.	The yield of oil via SC-CO_2_ extraction: 11.03–40.87; Solvent extraction: 48.48 to 56.13. Omega 3 PUFA content SC-CO_2_-extracted oils: 18.97 to 20.14; solvent-extracted oils: 20.37 to 21.38%.	[103]
Tuna (*Katsuwonus pelamis*)	Liver	Enzymatic extraction (EE), wet reduction (WR), SC-CO_2_ extraction method, and subcritical dimethyl ether (SDE) extraction.	Oils obtained with EE: 85.25 ± 1.29; WR: 56.76 ± 1.57; SC-CO_2_: 98.45 ± 1.04; and SDE: 98.57 ± 0.60. PUFA content of EE oil: 29.41 ± 0.16; WR: 29.31 ± 0.19; SC-CO_2_: 32.77 ± 0.19; and SDE: 32.83 ± 0.16.	[104]
Horse mackerel (*Trachurus mediterraneus*), seabream (*Pagellus acarne*), blue whiting (*Micromesistius poutassou*), and sardine (*Sardina pilchardus*).	Discards/by-products	Pre-heated fish discards at 40 °C for 30 min, and then discards were hydraulically pressed (120 bar) and centrifuged to recover the crude oils.	Yield of the oil, HM: 1 to 6.2; SB: 4.7 to 5.8; BW: 1.1 to 3.2; Sar: 2.5 to 18.8. PUFA content, HM: 35 to 43.1; SB: 37.1 to 44.7; BW: 26.3 to 38.9; and Sar: 39.6 to 42.6.	[105]
Japanese Spanish mackerel (*Scomberomorus niphonius*)	Skin, muscle, bone, head, and viscera	SC-CO_2_ extraction. Temperature: 45 °C; Pressure: 250 bar; Extraction time: 3 h.	Oils obtained—skin: 42.79 ± 1.79; muscle: 24.18 ± 1.09; bone: 29.11 ± 1.81; head: 31.08 ± 2.05; and viscera: 22.70 ± 1.35. PUFA content—skin: 27.54; muscle: 29.15; bone: 18.34; head: 21.88; viscera: 21.88.	[23]
Australian rock lobster (*Jasus edwardsii*)	Liver	SC-CO_2_ extraction method. Temperature: 50 °C; Pressure: 350 bar; Extraction time: 4 h.	Oil obtained: 24.3% (*w*/*w*); PUFA content: 31.3.	[106]
Conger eel (*Conger myriaster*)	Skin	SC-CO_2_ extraction method. Temperature: 55 °C; Pressure: 300 bar; Extraction time: 2 h.	Crude lipid: 71.9 ± 0.12 PUFA content: Omega 3: 18.62 ± 0.08; Omega 6: 4.16 ± 0.19.	[55]
Frigate tuna (*Auxis thazard*), Eastern little tuna (*Euthynnus affinis*), and Longtail tuna (*Thunnus tonggol*).	Viscera, skin, and heads	SC-CO_2_ extraction method and solvent extraction methods.	Crude oils obtained—viscera: 13.5–16.8; skin 21.8–26.4; and head 30.2–36.2. PUFA content: 24.1–27.9 where docosahexaenoic acid (DHA) was prominent.	[107]
Brazilian red-spotted shrimp (*Farfantepenaeus paulensis*)	Shell, tail, and heads	SC-CO_2_ extraction method. Temperature: 40–60 °C; Pressure: 200–400 bar.	Methods were reported about 4.9 ± 0.1% of oils obtained and optimized for carotenoid-rich oil extraction. PUFA content: EPA: 3.44 to 11.69; DHA: 2.25 to 12.20.	[108]
Northern shrimp (*Pandalus borealis*)	Shell, tail, and heads	SC-CO_2_ extraction. Temperature: 40 °C; Pressure: 350 bar.	Crude oils obtained—13.7 PUFA content—EPA: 7.8 ± 0.06; DHA: 8.0 ± 0.07.	[109]
Brown seaweeds (*Saccharina japonica* and *Sargassum horneri*)	Whole body	SC-CO_2_ extraction. Temperature: 45 °C; Pressure: 250 bar; Extraction time: 3 h.	Oil content: SJ: 1.09 ± 0.56; SH: 1.41 ± 0.15. PUFA content: SJ: 14.67; SH: 26.7.	[110]

### 3.4. Fishery By-Products as a Source of Minerals

Bones and shells obtained from fishery by-products are excellent sources of minerals and micronutrients [24]. Several studies have investigated the extraction of bone powders from various fish species and their nutritional properties. Fish frames are obtainable in two forms: cooked and uncooked. The uncooked frame (bone) is obtained from industry filleting, while the cooked bone is available in restaurants (containing collagen) after thermal treatment [111]. Calcium and phosphorus are the two major minerals obtained from the fish frames. Calcium plays a vital role in our body by facilitating different physicochemical activities, including strengthening neurological functions, bone and tooth health, and acting as a cofactor in many enzymatic reactions [112]. Calcium deficiency may cause osteoporosis and hypocalcemia, whereas phosphorus deficiency may cause a problem in metabolism. Fishbone contains approximately 60% calcium and 35% phosphorus [113]. Therefore, several methods have been developed to extract minerals from fishery discards. Fishbone powder has long been used for food fortification. Furthermore, minerals can maintain the textural properties of fortified foods. Fishery discards like fish bones are excellent sources of hydroxyapatite, which can be used as bone grafting materials [114] and in dental treatments [24,115]. Table 5 shows the extraction of minerals from different fishery by-products and their potential uses.

### 3.5. Seafood Wastes as a Source of Pigments

The beautiful coloration of different seafood species, such as fish, crustaceans, mollusks, and seaweeds, is due to the presence of various coloring agents commonly known as carotenoids [3]. Natural carotenoids in seafood by-products have strong health-beneficial activities [127]. The common carotenoids obtained from seafood by-products are astaxanthin, cantaxanthin, zeaxanthin, β-carotene, etc. [127]. Although carotenoids have biological activities, astaxanthin (3,3′-dihydroxy-β, β′-carotene-4,4′-dione) (red–pink color) has anticancer, neuroprotective, and strong antioxidant activities [14]. Supposedly, astaxanthin has 500 times more antioxidant activity compared with tocopherol (Vit-E) [128]. Astaxanthin is also beneficial for improving cardiovascular health and regulating blood pressure. Owing to their highly beneficial activities, marine carotenoids are used in food products, pharmaceuticals, and cosmetics [127]. Several carotenoids are used as preservatives in cosmetic products like ions and sun-protection creams [3]. Various carotenoids like α and β-carotene are the precursors of vitamin A and are considered trace elements for normal growth, immunological function, and vision [129]. Recently, carotenoid supplements for human consumption have become available on the market; hence, consumers are familiar with these products. The demand for marine carotenoids is booming and attracting the attention of scientists and industrialists [130].

In addition to carotenoids, other pigments are mainly obtained from marine algae, commonly known as phycobiliproteins. The major classes of phycobiliproteins are phycoerythrin (commonly found in red algae), phycocyanin (available in brown seaweed), and allophycocyanins [131]. Phycoerythrin has high antioxidant and anticarcinogenic activities and various health-beneficial activities [132].

There are various carotenoid extraction methods. An old method is the solvent extraction method. However, due to the drawbacks of solvent extraction [133], scientists are looking for green extraction methods such as SC-CO_2_ extraction [15,108], ionic liquid and deep eutectic solvent extraction [134], ultrasound-assisted extraction [15], microwave-assisted extraction [135], enzymatic hydrolysis [136], and extraction by various edible oils [89]. Owing to availability and market demand, astaxanthin extraction methods are highly established. Table 6 presents the extraction of the different pigments.

### 3.6. As a Source of Important Enzymes

Because of their lower side effects and higher bioactivities, enzymes are considered essential biomolecules [3]. Enzymes can work as catalytic agents for different reactions and, thus, reduce the cost of producing various compounds in different industries [137]. Enzymes from fishery by-products can be divided mainly into proteolytic and lipolytic enzymes [3]. Fishery by-products, especially viscera and head, are important sources of enzymes such as proteases and lipases [137]. Proteases are considered the most important class of enzymes, accounting for approximately 60% of the total enzyme market [3]. They are important for various food, pharmaceutical, and cosmetic applications. The fish viscera’s most important protease enzymes are pepsin, trypsin, chymotrypsin, and elastase [137]. Table 7 shows the enzymes available in fishery discards, their extraction methodologies, and their potential uses.

Fish enzymes have versatile applications in various industries. Protease enzymes play a vital role in the fish body during postmortem activities such as textural changes and flavor and trigger spoilage activities [138]. Another enzyme, serine protease, causes the black discoloration of shrimp by forming polyphenol oxidase [139]. In the industrial sector, proteases are used to extract valuable compounds from different raw materials via selective breakdown and can be used as substitutes for various biomolecules [3]. Lipolytic enzymes from seafood by-products are used to cleave the long chain of unsaturated fatty acids and prepare biodiesel [140]. Recent studies have reported that lipases extracted from seafood can be used to improve the flavor and odor of dairy products [141]. Several other enzymes also have commercial importance. Among them, chitinases [142], collagenases [143], and transglutaminases (TGase) [144] obtained from marine sources have notable industrial and medicinal importance. TGases isolated from different seafoods have been shown to improve the textural properties of various food products [145]. In addition to these mentioned enzymes, several other enzymes, such as ureases, alkaline phosphatase, phospholipases, alginate lyases, and xanthine oxidase, can also be extracted from fishery by-products [3].

**Table 6:** Extraction of natural pigments from marine fishery discards (types of fishery by-products used, extraction methodologies, types of pigments, and yield).

Fish Species	Parts of Body	Extraction Methods	Type of Pigments	Yield	References
Crabs, shrimp (*Penaeus indicus*), crayfish, krill, and lobster	Carapace and heads	Enzymatic hydrolysis with Trypsin (2000 U/g), papain (6000 NF Units), and alcalase (0.6 Anson U/g)	Crude carotenoids	Highest yield by alcalase 28.6 μg/g waste; papain (24.8 μg/g); and trypsin (25.3 μg/g).	[136]
Freshwater crab (*Potamon potamon*) and marine crab (*Charybdis cruciata*)	Shells and meat	Extracted by solvent extraction with acetone and ether	Astaxanthin, zeaxanthin, β-carotene	From the shell and meat of marine crab, astaxanthin was estimated about 65.5 and 67.6 g/100 g of carotenoids. From the shell and meat of marine crab, zeaxanthin was estimated about 0.49 and 5.0 g/100 g of carotenoids. From the shell and meat of marine crab, astaxanthin was estimated about 36.5 and 14.7 g/100 g of carotenoids. Zeaxanthin from shell and meat of freshwater crab about 74.8 and 42.0 g/100 g of carotenoids. The highest β-carotene was obtained from the meat of the freshwater crab, 7.4 g/10 g of carotenoids.	[146]
Spiny lobster (*Panulirus japonicas*)	Carapace	Acetone extraction	Canthaxanthin, astaxanthin, zeaxanthin, β-carotene, and adonixanthin	Total carotenoid yield: 0.1 mg/g carapace; Canthaxanthin: 6g/100 g of carotenoids; Astaxanthin: 65 g/100 g of carotenoids; Zeaxanthin: 1.2 g/100 g of carotenoids; β-carotene: 2g/100 g of carotenoids; Adonixanthin: 1.2 g/100 g of carotenoids.	[147]
Shrimp (*Peneanus monodon*)	Shells	Concurrent SC-CO_2_-extraction methodology	Astaxanthin-rich oil	A new process design for extraction of astaxanthin has been proposed and the highest yield obtained was 43.09 µg/g of oil.	[14]
Jumbo squid (*Dosidicus gigas*)	Skins	Solvent extraction with acidified methanol	Crude natural pigment	580 and 690 mg of pigment extract per 100 g of fresh squid skin.	[148]
White shrimp (*Litopenaeus vannamei*)	Hepatopancreas	Alkaline and heat treatment (1.0 M NaOH and pre-incubated at 60 °C)	Carotenoproteins	Carotenoproteins contained—73.58% protein and major carotenoids identified as astaxanthin and β-carotene.	[149]
Shrimp (*Peneanus monodon*)	Shells	Ultrasound-assisted natural deep eutectic solvent	Astaxanthin	Optimized the extraction methodology using response surface methodology, and the highest yield of astaxanthin was obtained at 68.98 ± 1.22 μg ASX/g shrimp waste.	[15]
Shrimp (*Penaeus vannamei*)	By-products	Ultrasonic-assisted ionic liquid extraction	Astaxanthin	Astaxanthin yield: 32.47 µg/g waste.	[150]
Red shrimps (*Aristeus antennatus*)	By-products	Ultrasound and microwave-assisted natural deep eutectic solvent	Astaxanthin	Ultrasound-assisted extraction: 7.85 ± 0.47 mg of astaxanthin/100 g dry sample; Ultrasound-assisted extraction: 26.7 ± 2 mg of astaxanthin/100 g dry sample.	[151]
Brown crab	Shell residues	Terpene-based natural deep eutectic solvents	Astaxanthin	The highest yield of astaxanthin was obtained at 9.3 ± 0.8 μg/g dry residue.	[152]
Northern shrimp (*Pandalus borealis*)	By-products	Sunflower oil (SF) and its methyl ester (ME-SF)	Astaxanthin	Yield obtained with SF: 23 mg/kg waste; ME-SF: 34.2 mg/kg waste.	[153]
Red microalgae (*Porphyridium* spp.)	Seaweed	Conventional extraction (maceration and freeze–thaw); Green extraction: (microwave (MW) and ultrasound (US)).	Phycoerythrin	The highest yield by maceration is 15.93 mg/g biomass; freeze–thaw was 16.08 mg/g biomass. Microwave: 23.94 mg/g biomass; ultrasound: 32.63 mg/g biomass.	[154]

**Table 7:** Enzymes extracted from different species of fishery by-products and their application.

Fish Species	Body Parts	Group and Name of the Enzymes	Application of Extracted Enzymes	References
Pink shrimp (*Parapenaeus* *longirostris*)	Gut, viscera and intestine.	Polyphenoloxidase -laccase	This enzyme exhibits very intense activity, and during storage, melanosis may continue to occur due to the oxidation of p-dihydroxyphenols produced mainly by non-specific hydroxylation of aromatic amino acids.	[139]
Leather jacket (*Aluterus monoceros*)	Pyloric caeca	Protease—trypsin	Preparation of protein hydrolysates with higher antioxidant activities.	[155]
New Zealand hoki (*Macruronus novaezealandiae*) and chinook salmon (*Oncorhynchus tshawytscha*)	Liver and intestine	Digestible lipases	Flavor development in dairy cream with extracted lipases and compared with calf pre-gastric esterase.	[141]
Goby (*Zosterisessor ophiocephalus*)	Viscera	Alkaline protease—crude extract	Deproteinization of shrimp wastes by extracted crude proteases.	[156]
Whiteleg shrimp (*Litopenaeus vannamei*)	Muscle, pleopods, digestive gland, and uropods	Lipase	Potential role in the hydrolysis of triacylglycerides stored as fat in the shrimp body.	[157]
Sardinelle (*Sardinella aurita*)	Viscera	A novel aspartic protease	Proteolytic activity was examined against natural food proteins.	[158]
Silver mojarra (*Diapterus rhombeus*)	Viscera	Alkaline peptidase—trypsin	With high activity and stability at pH from 8.5 to 11, this enzyme has good potential to be used as an additive in commercial detergent formulations.	[159]
Crayfish (*Pacifastacus leniusculus*)	Discards	Trans-glutaminase (TGase)	Extracted crayfish TGase enzyme showed higher activity at low temperatures (4 °C) than pig liver TGase.	[160]
Tilapia (*Oreochromis mossambicus*), bigeye snapper (*Priacanthus hamrur*), common carp (*Cyprinus carpio*) and Indian oil sardine (*Sardinella longiceps*)	Fish muscle tissue	Trans-glutaminase (TGase)	Improvement of the setting and gelling ability of fish mince from *Cynoglossus* spp.	[144]
Antarctic krill (*Euphausia superba*)	By-products	Trans-glutaminase (TGase)	Extracted enzymes enhanced the mechanical properties of gelatin gels at 4 °C.	[161]

## 4. Macromolecules Obtained from Fishery By-Products

### 4.1. Gelatin and Collagen

Collagen proteins are obtained from fish by-products such as bones, skin, and cartilage and are found in the tissues of both vertebrates and invertebrates [162]. Both collagen and gelatin are highly used in the food, pharmaceutical, and cosmetics sectors. The animal body is claimed to contain approximately 30% collagen of its total protein component [163]. Due to the religious aspect, marine collagen is highly acceptable to Muslim- and Hindu-based countries due to the religious restriction for consuming porcine and bovine collagen. Interest in marine collagen is expanding daily among scientists and industrialists due to its high demand and unique physicochemical properties, and it will reach a market value of 1055.2 million USD by 2026 [3].

The collagen molecule has a triple helix structure and comprises three polypeptide chains, and its molecular weight is approximately 100 kDa [164]. Collagen molecules can be subdivided into three types based on cross-linking intensity. Type-I collagen is available in all collective tissues, type-II is primarily found in cartilages, and type-III is mainly found in body parts such as the intestine [165]. Gelatin can be obtained via the heat treatment of collagen [164]. Gelatin obtained from marine fishery discards has lower rheological properties such as melting point and viscosity; however, it has good homeostatic properties compared with mammalian gelatin and better metabolic compatibility [166].

Marine teleosts are considered important sources of collagen as they contain 75% of their total body weight [162]. Marine fishery discards, particularly fish skin, scales, and bones, are sustainable and cost-effective sources of collagen. As the interest in marine collagen is increasing, several scientists have extracted collagen from fishery by-products such as cod skin [167], fish scales [168], tuna skin and scales [169], squid fins and arms [170], and salmon scales and skin [171]. Collagen from marine fishery by-products is mainly extracted using acid solubilization. However, different enzymes, such as collagenase, can also be used to target this valuable macromolecule [172]. The collagen yield highly depends on the species and can vary from 1% to 50% of the raw materials [3].

Collagen has been used in the food, cosmetic, and pharmaceutical industries. Purified collagen has been used for tissue engineering by preparing different biomaterials such as gels, scaffolds, sponges, and nanocomposites [173]. Gelatin is used for preparing biodegradable packing films and nanoparticles, microencapsulating various bioactive compounds, and tuning the texture properties of foods [174]. Table 8 shows recent studies on the extraction of collagen, gelatin, and their derivatives.

**Figure 4:**
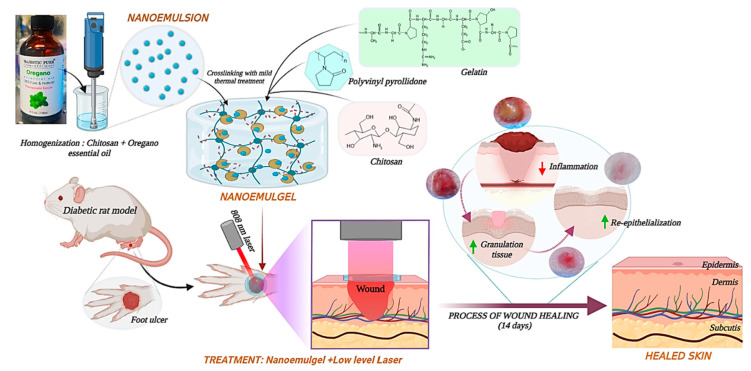
Chitosan-gelatin-based hydrogel loaded with drugs has been applied to treat diabetic foot ulcers. “Adapted with permission from Razack et al. [188]. 2022, Elsevier B.V.”.

As previously mentioned, collagen has a huge potential in the pharmaceutical industry [175]. Due to its biocompatibility and biodegradability, collagen and its derivatives are widely used in different medicinal products. A recent study showed the potential of collagen-based dressing for drug delivery as an attractive and promising system in medical applications [176]. Collagen or gelatin and their derivatives can also be used for tissue engineering and quick wound healing (Figure 4). It has also been reported that collagen scaffolds can play an important role in bone and cartilage reconstruction [177]. Different facial creams prepared with collagen are popular due to their activity [162,178].

### 4.2. Seafood By-Products as Sustainable Sources of Polysaccharides

Seafood discards, especially the shells of crustaceans, exoskeletons of mollusks, and seaweeds, are sources of various polysaccharides [179]. The utilization of crustacean by-products is challenging because they contain approximately 75% of discards, are not easily decomposed in nature, and are considered an environmental hazard [180]. Chitin and chitosan extraction from crustacean shells are well-known, and they have a long history of application in the food, pharmaceutical, and cosmetic industries [179]. Chitin is a highly available polysaccharide after cellulose, which comprises 1,4-poly-N-acetyl-D-glucosamine. The most exciting information is that approximately 100 billion tons of chitin can be generated from nature if we can use all the resources, such as insects, crustacean shells, fungi, and all other organisms [181]. According to several studies, the market value of chitin was 6.8 billion USD in 2019, and by 2027, its market value is expected to increase by 24.7%, indicating its necessity in various industries [182]. By deacetylating chitin, another popular polysaccharide, chitosan (poly-β-1,4–2-amino-2-deoxy-D-glucopyranose), can be generated [183]. This polymer is well-known for its diverse applications in various fields. It is biodegradable [184], nontoxic [183], and has multiple biofunctional activities such as antioxidant [185], antimicrobial [186], antifungal [187], anticancer [183], and wound healing activities [188]. This functional biomolecule also helps lower cholesterol levels [189], acts as an anti-inflammatory agent [190], and inhibits tumor formation [191]. The diverse applications of chitosan also include biodegradable packaging, nanocomposite preparation, drug delivery, the preparation of various hydrogels, and therapeutic agents. Low-molecular-weight chitosans or the oligomers of chitins and chitosans are called chitooligosaccharides [182]. They are highly soluble in water and have better functionality than native chitin and chitosan [183].

Chitin extraction through conventional processes requires high heat and various chemicals. Chitin extraction usually has three steps: (i) demineralization, which requires the application of strong acids, including HCl, HNO_3_, and H_2_SO_4_; (ii) deproteinization by alkaline treatment using NaOH or KOH; and (iii) decolorization by organic solvents. However, after chitin extraction, an extra step called deacetylation by alkaline treatment using strong NaOH or KOH is necessary [183]. Due to the application of synthetic chemicals during chitin and chitosan extraction, they impose hazardous effects on the environment; scientists are looking for different green and eco-friendly approaches to extract these valuable molecules. Recently, several green extraction methods have been used to extract chitin and chitosan. They include the application of bacterial fermentation [192], enzymatic hydrolysis [193], subcritical water [194], ultrasound [195], microwave-assisted [196], ionic liquid [197], deep eutectic solvents [198], and pulsed electric field extraction [199].

Fucoidan is an important sulfated polysaccharide available in marine algae (brown seaweed) and different invertebrates such as sea cucumbers and sea urchins [200]. Researchers have been very interested in this marine polysaccharide because of its biological potential since it was discovered in 1913 [200]. It is negatively charged and hygroscopic [201]. The solubility of this polysaccharide in both water and acid solutions makes it a suitable candidate for medicinal applications over other sulfated polysaccharides [202]. In recent decades, fucoidans isolated from marine sources have been extensively studied to discover their potential use in the medical sector. Fucoidans have strong antioxidant, anticoagulant, antitumor, and antiviral activities [203]. They are also effective in improving the digestive and urinary systems [204]. The main constituents of fucoidan are fucose and sulfate. However, the chemical structure also contains different monosaccharides such as glucose, galactose, and other compounds such as uronic acid [200].

**Figure 5:**
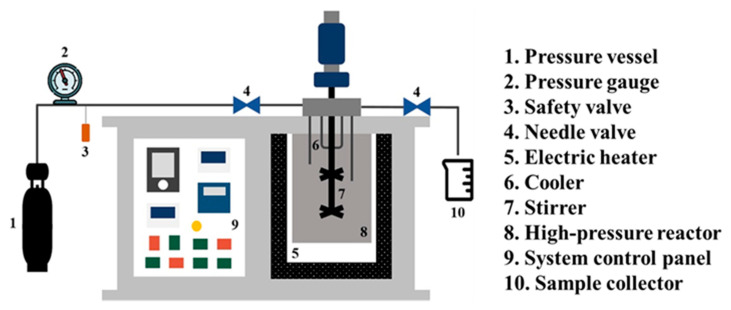
Subcritical water hydrolysis is a promising and alternative green extraction method to obtain polymers and hydrolysates from seafood by-products. This figure has been adapted from our previous study by Lee et al. [56].

Fucoidan extraction via conventional methods, such as dilution in acetic acid, has a long history [203]. Crude fucoidan extracted via conventional methods often contains different contaminants, requiring purification. Column chromatography is a popular technique for purifying fucoidan [204]. Recently, various green extraction methods have been applied to extract fucoidan, such as subcritical water extraction [205] (Figure 5), ultrasound-assisted extraction [206], and microwave-assisted extraction [207]. Table 9 shows recent studies on the extraction of polysaccharides, their extraction methods, and the functionalities of the extracted polysaccharides.

**Table 8:** Extraction of collagen and gelatin and their derivatives, their functionalities, and their potential applications from various fishery discards.

Fish Species	Body Parts	Type of Collagen/Gelatin	Yield	Properties/Finding of the Study	References
Bullhead shark (*Heterodontus japonicas*), Ayu (*Plecoglossus altivelis*), Horse mackerel (*Trachurus japonicas*), Skipjack tuna (*Katsuwonus pelamis*), yellow sea bream (*Dentex tumifrons*), chub mackerel (*Scomber japonicas*), and Japanese sea-bass (*Lateolabrax japonicas*)	Fins, bones, and skins	Acid-solubilized collagen (ASC)	(1) Skin collagen, 51.4% (Japanese sea-bass), 49.8% (chub mackerel), and 50.1% (bullhead shark), respectively; (2) bone collagen, 42.3% (skipjack tuna), 40.7% (Japanese sea-bass), 53.6% (ayu), 40.1% (yellow sea bream), and 43.5% (horse mackerel), respectively; (3) fin collagen, 5.2% (Japanese sea-bass acid-soluble collagen) and 36.4% (Japanese sea-bass acid-insoluble collagen).	This report indicates that these fish waste materials have the potential to supplement the skin of land vertebrates as a source of collagen.	[208]
Spanish mackerel (*Scomberomorous niphonius*)	Skin	Acid-solubilized collagen (ASC)	The collagen obtained from the skin is 13.68 ± 0.35%.	Antioxidant activities; emulsifying properties of the extracted collagen varied by average molecular weight.	[209]
Jumbo squid (*Dosidicus gigas*)	Skin and fins	Extraction of acid-solubilized collagen (ASC) and then enzymatic hydrolysis	Collagen was obtained—from fin: 69%; and from skin: 66% (based on dry wt.).	Extracted collagen showed higher levels of polar and hydrophobic amino acids. The collagen hydrolysates produced by subtilisin showed a lower degree of hydrolysis and higher antioxidant activity.	[170]
Smooth-hound (*Mustelus mustelus*)	Skin	Acid-solubilized collagen (ASC) and pepsin-solubilized collagen (PSC)	Collagen obtained—ASC (acid-soluble): 23.07%; and PSC (pepsin-soluble): 35.27% of the sample.	Extracted collagen used for preparing films with chitosan and prepared biofilm showed potential UV barrier properties and antioxidant activity.	[210]
Red drum fish (*Sciaenops ocellatus*)	Scales	Acid-solubilized collagen (ASC) and pepsin-solubilized collagen (PSC)	Collagen obtained—ASC (acid-soluble): 0.61 ± 0.20%; and PSC (pepsin-soluble): 4.32 ± 0.30% of the sample.	Type-I collagen was isolated rapidly via hydrophilic ultrafiltration from the scales of red drum fish (Sciaenops ocellatus) after limited pepsin digestion.	[168]
Bigeye tuna (*Thunnus obesus*)	Bones, scales, and skin	Acid-solubilized collagen (ASC) and pepsin-solubilized collagen (PSC)	Collagen was obtained from skin—ASC (acid-soluble) 13.05 ± 0.6%; and PSC (pepsin-soluble) 16.7 ± 0.7% based on dry wt. Collagen was obtained from scale and bone—PSC (pepsin-soluble) 4.6 ± 0.3% and 2.6 ± 0.3% based on dry wt.	This study concluded physiochemical properties of extracted fish collagen were comparable to mammalian collagen.	[169]
Black ruff (*Centrolophus niger*)	Skin	Acid-solubilized collagen (ASC)	The yield of the extracted collagen varied from 25% to 45% based on the skin weight.	Extraction and characterization of collagen from fish waste and its application in the development of antibacterial active food-packaging film.	[211]
Atlantic cod and Atlantic salmon	Scales and skin	Acid-solubilized collagen (ASC)	Yield of collagen—Atlantic salmon: skin 11.95% and fins: 5.76%. Atlantic cod: skin: 3.46% and fins: 4.34% based on wet tissue.	Salmon scales and skin had very high collagen levels, allowing them to be promising sources for high-value collagen production.	[171]
Eel fish (*Evenchelys macrura*)	Skin	Acid-solubilized collagen (ASC) and pepsin-solubilized collagen (PSC)	Collagen yield—ASC: 80% and PSC: 7.1% based on the dry weight of the skin.	The ASC and PSC gels and films also showed equal potency in delivering drugs against bacterial and fungal human pathogens.	[212]
Blue whiting (BW, *Micromesistius poutassou*), Mackerel (M, *Scomber scombrus*), Red scorpionfish (RS, *Scorpaena scrofa*), and Pouting (P, *Trisoreptus luscus*)	Heads and skins	Extraction of gelatin using a sequential combination of 0.05 M NaOH, 0.02 M H_2_SO_4_, and 0.05 M citric acid solutions.	Yield of the gelatin (%. *w*/*w* fresh skin)—BW: 0.23 ± 0.05; M: 0.69 ± 0.33; RS: 0.28 ± 0.11; P: 0.56 ± 0.25.	Extracted gelatin showed strong antioxidant and antihypertensive activity.	[10]
Atlantic mackerel (*Scomber scombrus*)	Skin	Extraction of gelatin via acid-based and heat treatment	Gelatin yield varied from 29.6 to 31.8%.	The chemical composition, rheological and textural properties, and microstructural characteristics of the extracted gelatins were analyzed and compared with commercial bovine hide gelatin.	[213]
Mackerel (*Scomber japonicus*)	Bone and skin	Collagen hydrolysate using subcritical water hydrolysis	Subcritical water treatment produced low-molecular-weight (<1650 Da) collagen peptides.	The antioxidant activities of collagen hydrolysate obtained via subcritical water hydrolysis were significantly higher than native collagen.	[214]
Bigeye tuna (*Thunnus obesus*)	Bones, scales, and skin	Collagen hydrolysate via catalyst-assisted subcritical water hydrolysis	The average molecular size of the peptides in the obtained collagen hydrolysates varied between 300 and 425 Da.	The collagen hydrolysates obtained in this study showed enormous potential for use in the food and pharmaceutical industries.	[215]
Totoaba (*Totoaba macdonaldi*)	Swim bladder	Pepsin-solubilized collagen (PSC) and collagen hydrolysates via enzymatic hydrolysis (Alcalase and papain)	The yield of collagen was high (68%) and exhibited good thermal stability (32.5 °C).	This study reported that the swim bladder from the farmed totoaba could be an ideal source to produce high-quality type-I collagen and may be considered an alternative to conventional collagen sources.	[216]

**Table 9:** Conventional and green extraction methods for extracting polysaccharides from marine fishery discards and their functionalities.

Seafood	Body Parts	Extraction Methods	Polysaccharide	Yield	Properties/Characteristics	References
Pacific white shrimp (*Litopenaeus vannamei*)	Heads	Demineralization and deproteinization through HCl and NaOH solutions. The deacetylation process obtained chitosan	Chitin and chitosan	Chitin and chitosan were obtained from shrimp waste processing about 25 ± 2 g/kg and 17 ± 4 g/kg.	Anticoagulant properties and anti-inflammatory activity.	[217]
Shrimp	Shrimp waste	Chitin extracted with conventional methods. Chiton extracted using microwave-assisted extraction	Chitin and chitosan	The maximum yield obtained from shrimp waste was about 36.43% (based on dry wt.), and the highest chitosan yield was 90% based on the chitin wt.	Antibacterial, functional, antioxidant, and physicochemical properties.	[218]
Shrimp (*Metapenaeus monoceros*)	Shells	Enzymatic extractions by several microbial and fish alkaline proteases	Chitin and chitosan	Concerning microbial enzyme preparation, high deproteinization (DDP) degrees were obtained with 77 ± 3%.	Antimicrobial, antitumor, and antioxidant activities.	[219]
Norway lobster (*Nephrops norvegicus*)	Thorax, heads, and appendix by-products	Enzymatic extraction (protease from *Bacillus lentus*)	Chitin	The yield of the chitin extracted from Norway lobster was 24.6 ± 1.02% (based on dry wt.).	Antiproliferative and antimicrobial activities.	[220]
Shrimp (*Marsupenaeus japonicas*)	Shells	Deep eutectic solvent extraction	Chitin	A higher yield was obtained compared with the conventional extraction (16.08%).	Extracted chitins showed excellent potential for preparing biodegradable packaging film.	[198]
Lobster	Shells	DES (Deep eutectic solvent)	Chitin	The highest yield of chitin was 23.31% with Choline chloride-lactic acid deep eutectic solvent.	Acid-based deep eutectic solvents have the potential for use as green media for the production of chitin.	[12]
Shrimp	Shells	Extraction by ammonium-based ionic liquids	Chitin and chitosan	A chitin extraction of 14% of the original biomass was found after shrimp-shell treatment with ionic liquids.	The experimental results revealed that ionic liquids could be a potential medium for chitin extraction.	[197]
Prawn	Shells	Microbial extraction (fermentation)	Chitin and chitosan	The highest yield of chitin was 0.78%, with a higher degree of deacetylation of 72.90%.	A higher degree of deacetylation is valued compared with the commercial chitin.	[221]
Shrimp	Shells	Ultrasound-assisted extraction	Chitin and chitosan	Ultrasound reduces the protein content and particle size of chitin.	Chitosan of high deacetylation and medium molecular weight was produced, and the extracted chitosan was applied for beef preservation.	[222]
Swimming crab (*Portunus trituberculatus*)	Shells	Subcritical water pretreatment	Chitosan	The yield and the molecular weight of the chitosan were 12.2% and 1187.2 kDa, respectively.	Chitosan prepared via subcritical water pretreatment was easier to use in preparing oligosaccharides.	[223]
Shrimp (*Penaeus monodon*)	Shells	Subcritical water	Oligochitosan	Subcritical water hydrolysis reduces the molecular weight of the chitosan (3.06 kDa).	Oligochitosan showed potent antioxidant, antimicrobial, and anticancer activities.	[183]
Shrimp	Shells	Fermentation by *Pseudonocardia antitumoralis*	Chitooligosaccharides	The results indicate that the isolate *Pseudonocardia antitumoralis* 18D36-A1 could convert chitin into chitooligosaccharides.	The extract produced the active fraction D36A1C38, which can inhibit the growth of fungi by 74% at a concentration of 1 mg/mL.	[224]
Shrimp	Discards	Co-fermentation in the presence of *Bacillus subtilis* and *Acetobacter* sp.	Chitooligosaccharides	Final deproteinization (DP) and demineralization (DM) efficiency and the chitin yield were achieved as 94, 92, and 18%.	The proposed method exhibited excellent stability and high hydrolysis efficiency.	[225]
Brown algae (*Turbinara ornata*)	Seaweed powder	Conventional methods in acid dilution	Fucoidan	10 different fractions of the crude fucoidan were obtained, and the highest sulfate content was reported as 38.34%.	This study claimed to be the first report to illustrate the potential anti-inflammatory activity of fucoidan extracted from the brown algae *T. ornata.*	[204]
Brown seaweed (*Saccharina japonica*)	Seaweed powder	Subcritical water extraction (SWE) with different solvents	Fucoidan	The highest yield of crude fucoidan was 8.23 at 140 °C, 50 bar, and 0.1% NaOH solvent.	A high yield of fucoidan was obtained from SWE when compared with the conventional method, and crude fucoidan showed high antioxidant and emulsifying activity properties.	[205]
Brown seaweed (*Fucus vesiculosus*)	Seaweed powder	Microwave-assisted extraction (MAE)	Fucoidan	The highest yield of fucoidan was 15.61%, and its sulfate content was 22.76%.	This method required short extraction times and non-corrosive solvents, resulting in reduced costs for green extraction techniques.	[207]

## 5. Conclusions and Implication of This Review Work

This study summarized the presence of valuable bioactive compounds and their functionalities. In recent decades, the massive production of fishery products has introduced enormous fish discards. Some are effectively used in agriculture (fertilizers), animal foods, pharmaceuticals, and cosmetics. However, most discards are disposed of in the sea or other water bodies and negatively impact environmental status. Thus, they need to be managed with effective fishery management. Valorizing fish by-products for industries is challenging, and the necessary techniques should be taken. Most methods are costly and challenging to manage.

Caruso et al. [24] indicated that the losses of bioactive compounds from the by-products due to poor handling and dumping negatively affect the environment. Thus, to overcome these problems, the most effective techniques were used to extract or isolate bioactive compounds (e.g., lipids, proteins, chitin, enzymes, peptides, etc.) and use those valuable nutrients as food ingredients (with regulated food quality, production, distribution, and proper marketing). Enzymatic hydrolysis, SC-CO_2_ extraction, subcritical water extraction, and other green extraction methods can be applied for the complete valorization of these by-products. However, the development of techniques and methods to effectively valorize seafood discards is ongoing. We believe that this narrative study for an up-to-date understanding of fishery discards’ valorization will provide scientists and researchers with new insights into the extraction of valuable treasures from seafood discards. However, the application of bioactive compounds from fishery sources is increasing daily among health-conscious consumers. Food, pharmaceutical, and cosmetic products from several bioactive compounds, such as PUFA-containing fish oil, collagen, gelatin, and peptides, are available. Due to consumer demand, this field is flourishing and requires further and advanced research. It is believed that the ocean will be the source of various medicinal products in the future, as seafood already provides thousands of valuable bioactive compounds to humans.

To achieve a sustainable and hunger-free world, minimizing the discarding of food products is essential. From the food perspective, fishery by-products have tremendous potential for the blue economy and the initiative toward zero-waste management. More studies are required to develop an innovative approach to utilizing fishery discards and extracting their bioactive and nutritional compounds, which can ensure a circular economy in fishery-based industries.

## Figures and Tables

**Table 1:** Major by-products are obtained from various seafoods.

Groups	Name of the Seafood	By-Products		References
		Body Parts	Percentage (*w*/*w*)	
Mollusca	Octopus, squid, cuttlefish	Skin, heads, fins, tentacles, and guts	Up to 60	[17]
	Clam, oyster, scallop, mussel, etc.	Shells and viscera	60–80	[18]
Crustacean	Crayfish	Head and shell	Up to 80	[19,20]
	Shrimp	Head, shell, and tail	40–45	[14,15]
	Lobster	Heads, shells, livers, and eggs	50–70	[21]
	Crab	Viscera, shell	Up to 85	[22]
Finfish and other cartilaginous fishes		Trimming	2–5	[6,11,23]
		Skin	1–5	
		Scales	2–4	
		Bones	10–34	
		Liver and gut	15–20	
		Head	15–20	

**Table 5:** Extraction of fish minerals and their application in the preparation of various products for biomedical applications.

Species	Body Part	Extraction of Minerals	Final Products and Activities	References
Salmon (*Salmo salar*) and sea bream (*Sparus aurata*).	Bones	Alkaline treatment (NaOH) for 24 h; Calcination at 850 °C for 4 h.	Fishbone-derived biphasic calcium phosphate coatings with improved textural, anti-inflammatory, and antimicrobial properties.	[116]
Spotted sorubim (*Pseudoplatystoma corruscans*).	Bones	Cleaned and washed with hot water; Calcination with 900 °C temperature.	Nanocomposite with improved mechanical and physical properties.	[117]
Grey triggerfish (*Balistes capriscus*) and *Black* scabbardfish (*Aphanopus carbo*).	Skin and bones	Isolation of hydroxyapatite 400, 600, 800, 1000 °C temperature.	Preparation of natural biphasic materials for targeting bone grafting.	[113]
Salmon (*Salmo salar*), Red scorpionfish (*Scorpaena scrofa*), and Atlantic horse mackerel (*Trachurus trachurus*).	Bones	Biogenic calcium phosphate was obtained via alkaline (hydrolysis) treatment and calcination with 750, 900 °C temperatures.	Biphasic carbonated hydroxyapatite (HA)/beta-tricalcium phosphate (TCP) and their application in the biomedical field.	[118]
Cuttlefish (*Sepia* *Officinalis*).	Bones	Calcination with 700 °C temperature for 120 min.	Biphasic calcium phosphate scaffolds targeting bone tissue engineering applications.	[119]
Cuttlefish (*Sepia* *Officinalis*).	Bones	Boiling in water, dipped for 1 h; Calcination with 900 °C temperature for 240 min.	Synthesis of biphasic calcium phosphate for hydrogel sample preparation.	[120]
Tuna (*Thunnus thynnus*) and sword fish (*Xiphias gladius*).	Bones	Boiled and wasted water jet (strong) for 1 h; Calcination with 600, 950 °C temperature 12 h.	Biological hydroxyapatite for biomedical application.	[121]
Sardine, salmon (*Salmo salar*), and sablefish (*Anoplopoma* *Fimbria*).	Bones	Boiled for 2 h with deionized water and flowing water wasted; Calcination with 600–1100 °C temperature for 60 min.	HA/β-TCP biphasic calcium phosphate ceramics (BCP) are produced from fish bones.	[122]
Nile tilapia (*Tilapia nilotica*).	Scales	Distilled water wasted and dried up; Calcination with 800 °C temperature.	Obtained hydroxyapatite powder for preparing biphasic calcium phosphate coating.	[123]
Salmon (*Salmo salar*).	Bones	Boiled for 2 h with deionized water and flowing water wasted; Calcination with 600 °C temperature for 60 min.	Construction of HA/β-TCP biphasic ceramic as a novel bone graft material.	[124]
Nile tilapia (*Tilapia nilotica*).	Scales	Washed with 0.1 M HCl several times and dried at 60 °C after washing with distilled water. Afterwards, alkaline treatment with NaOH at 100 °C to obtain the hydroxyapatite.	Nanocrystalline hydroxyapatite and its application for selenium adsorption in aqueous solution.	[125]
Salmon (*Salmo salar*).	Bones	Boiled for 1 h with 1% of NaOH and pure water wasted; Calcination with 800 °C temperature for 180 min.	Preparation of calcium phosphate bioceramics for bone-substitute materials.	[126]

## Data Availability

All data are shown within the manuscript.
